# Simulating the dynamics of dispersal and dispersal ability in fragmented populations with mate‐finding Allee effects

**DOI:** 10.1002/ece3.10021

**Published:** 2023-04-21

**Authors:** Andrew J. Schauf, Matthew F. Jones, Poong Oh

**Affiliations:** ^1^ Department of Physics National University of Singapore Singapore Singapore; ^2^ NUS Cities National University of Singapore Singapore Singapore; ^3^ Biodiversity Institute University of Kansas Lawrence Kansas USA; ^4^ Department of Ecology and Evolutionary Biology University of Kansas Lawrence Kansas USA; ^5^ Biodiversity Knowledge Integration Center, School of Life Sciences Arizona State University Tempe Arizona USA; ^6^ Wee Kim Wee School of Communication and Information Nanyang Technological University Singapore Singapore

**Keywords:** Allee effect, dispersal, evolution of dispersal ability, fragmented population, mate finding, reaction–diffusion model

## Abstract

We consider the spatial propagation and genetic evolution of model populations comprising multiple subpopulations, each distinguished by its own characteristic dispersal rate. Mate finding is modeled in accord with the assumption that reproduction is based on random encounters between pairs of individuals, so that the frequency of interbreeding between two subpopulations is proportional to the product of local population densities of each. The resulting nonlinear growth term produces an Allee effect, whereby reproduction rates are lower in sparsely populated areas; the distribution of dispersal rates that evolves is then highly dependent upon the population's initial spatial distribution. In a series of numerical test cases, we consider how these dynamics affect lattice‐like arrangements of population fragments, and investigate how a population's initial fragmentation determines the dispersal rates that evolve as a habitat is colonized. First, we consider a case where initial population fragments coincide with habitat islands, within which death rates differ from those that apply outside; the presence of inhospitable exterior regions exaggerates Allee effect‐driven reductions in dispersal ability. We then examine how greater distances separating adjacent population fragments lead to more severe reductions in dispersal ability. For populations of a fixed initial magnitude, fragmentation into smaller, denser patches leads not only to greater losses of dispersal ability, but also helps ensure the population's long‐term persistence, emphasizing the trade‐offs between the benefits and risks of rapid dispersal under Allee effects. Next, simulations of well‐established populations disrupted by localized depopulation events illustrate how mate‐finding Allee effects and spatial heterogeneity can drive a population's dispersal ability to evolve either downward or upward depending on conditions, highlighting a qualitative distinction between population fragmentation and habitat heterogeneity. A final test case compares populations that are fragmented across multiple scales, demonstrating how differences in the relative scales of micro‐ and macro‐level fragmentation can lead to qualitatively different evolutionary outcomes.

## Graphical abstract

We model the reaction–diffusion dynamics of complex populations represented by multiple population density functions, each distinguished by its own characteristic dispersal rate, under the influence of mate‐finding Allee effects. In a series of numerical experiments, we simulate the evolution of dispersal ability in fragmented model populations, demonstrating how a population's initial spatial distribution affects the dispersal characteristics that evolve as it colonizes a region.

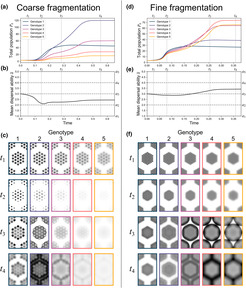

## 1 INTRODUCTION

### 1.1 Fragmentation and dispersal ability

Changes to a population's habitat can lead to changes in the phenotypic traits that the population exhibits. This is perhaps most visible through phenomena such as *island gigantism* and *island dwarfism* (Benítez‐López et al., 2021; Lomolino, 2005; McClain et al., 2013; Raia & Meiri, 2006; Van Valen, 1973). Aside from changes in size, insular populations can also evolve drastic morphological differences, such as those that lead to flightlessness in birds (Wright et al., 2016) or increased woodiness in plants (Lens et al., 2013). It has been suggested that most (if not all) of these evolutionary changes reflect the same consistent trend toward *reduced dispersal ability* on islands, or even beyond islands (Filin & Ziv, 2004; Lomolino, 2005; Waters et al., 2020; Whittaker & Fernández‐Palacios, 2007). Darwin famously pondered insular dispersal ability loss by considering an analogy with shipwrecked mariners facing a choice between clinging to the shipwreck or swimming away (Lomolino, 2009). Individuals with an innate tendency to stay put (i.e., slower dispersers) would remain, while those predisposed to swim away (i.e., faster dispersers) would leave. In this way, slower dispersers could come to consolidate themselves there, reducing the local population's dispersal ability. A variety of mathematical analyses repeatedly predicted similar tendencies toward dispersal ability loss, even beyond island habitats (Asmussen, 1983; Balkau & Feldman, 1973; Filin & Ziv, 2004; Hastings, 1983; Holt, 1985; Johnson & Gaines, 1990). The downward evolution of dispersal ability in these models is typically driven by the adverse effects suffered disproportionately by rapid dispersers as they traverse harmful features of their habitats, such as dangerous boundaries or gradients in environmental quality. In this way, these models typically assume that some form of *environmental heterogeneity*, rather than *spatial isolation*, is the primary factor shaping the evolution of dispersal characteristics. In doing so, they often draw conclusions which do not depend on the initial distribution of a population throughout its environment.

Elsewhere, spatial isolation *has* often been used to explain these insular phenomena, whether they occur on true islands bounded by water, or on *habitat islands*, where other forms of isolation lead to “island effects” such as body size change and dispersal ability loss (Amburgey et al., 2021; Cayuela et al., 2019; Haila, 2002; Incagnone et al., 2015; Lens et al., 2013; McClain et al., 2006; Merckx et al., 2018). The isolation of population fragments from one another has long been recognized to have a complex influence on the evolution of populations and species beyond islands as well (Kisel & Barraclough, 2010; Losos et al., 2010; MacArthur & Wilson, 2001; Whittaker et al., 2017; Whittaker & Fernández‐Palacios, 2007). Spatial isolation can alter the selective pressures that shape the evolution of a segment of a population (Jessop et al., 2018; Lomolino et al., 2012; McClain et al., 2013; Millien, 2004) by sheltering it from predators, competitors, and environmental hazards, or conversely by separating individuals from resources or from their own peers, *including potential mates*. Even within Darwin's “shipwreck” scenario, the prospects of survival for slower or faster dispersers would depend on the details of the shipwreck, including how the mariners and ship fragments were distributed through space relative to one another and to various features of the surrounding environment. In realistic populations and habitats too, spatial heterogeneity can result in a complex interplay between environments, populations, and the patterns of dispersal and dispersal ability that evolve.

Both habitats and the populations that inhabit them can simultaneously exhibit heterogeneity, patchiness, or fragmentation, with the distances between population fragments recognized as a factor in the likelihood of successful dispersal (Bowler & Benton, 2005; Conradt et al., 2000). Empirical observations of *genetic rescue*—by which migration‐driven gene flow reintroduces genetic diversity into isolated population fragments, supporting their continued adaptation and survival—demonstrate how flows between separate fragments can be mediated by individuals with high dispersal ability (Bell et al., 2019; Ingvarsson, 2001; Räsänen & Hendry, 2008; Whiteley et al., 2015). Natural or anthropogenic disturbances can alter habitats while also affecting population densities across affected areas, fragmenting habitats and populations; variations in body size and dispersal ability have been observed to follow these events (Brisson et al., 2003; Griffiths & Brook, 2014; Merckx et al., 2018; Palkovacs et al., 2012). When populations expand into unpopulated areas, dispersal abilities have been observed to evolve *upward* along the advancing edges of population fragments (Bénichou et al., 2012; Bouin et al., 2012; Deforet et al., 2019; Holt et al., 2004; Hughes et al., 2007; Léotard et al., 2009; Phillips et al., 2010; Travis et al., 2009). These examples highlight the potentially crucial role of heterogeneity of a population's distribution throughout an environment—*population fragmentation*, as distinct from *habitat fragmentation*—in shaping the evolution of dispersal‐related traits. This study thus focuses upon the lesser studied aspects of *population fragmentation*, in terms of which issues of spatial isolation can be disentangled from heterogeneity in the underlying habitat. Using a reaction–diffusion model, we demonstrate how the details of a population's initial fragmentation in space can play an important role in shaping the dispersal characteristics that develop when a genetically diverse population reproduces sexually. Before presenting the model, we will review related previous work on reaction–diffusion and metapopulation models of coupled spatial‐genetic dispersal dynamics, as well as mate‐finding *Allee effects*, by which spatial aggregation, rather than isolation, becomes advantageous for sexually reproducing populations.

### 1.2 Modeling dispersal and the evolution of dispersal ability

#### 1.2.1 Reaction–diffusion models

A population's spatial movements can change its patterns of exposure to its environment, while also affecting how frequently different types of individuals within the population interact with one another. These changes, in turn, alter the birth and death processes that shape the population's genetics, including those traits that determine how it moves through space. This results in a complex feedback between dispersal and reproduction that can be readily captured by *reaction–diffusion* equations. Skellam's (1951) pioneering reaction–diffusion model describes how a population, represented by a density function, evolves under simultaneous processes of random‐walk dispersal and reproduction (see Box 1, Equation B1). Considering a model habitat encircled by a “zone of absolute extinction,” Skellam concluded that a population with a slower dispersal rate would grow more quickly, while a population of faster dispersers would grow more slowly, or even decay, as it spilled more rapidly outward into the habitat's lethal exterior. Filin and Ziv (2004) later invoked this result to explain the apparent universal tendency toward dispersal ability loss on islands: subpopulations with slower dispersal rates would grow faster than subpopulations of rapid dispersers. However, this explanation relies on the assumption of passive dispersal across a lethal “absorbing” island boundary. Its heuristic arguments also overlook the possibility that subpopulations distinguished by different dispersal rates can mate, interacting through reproduction to potentially “rescue” one another from extinction. These limitations demonstrate the need for reaction–diffusion models that can (1) accommodate a wider range of domains and boundaries, and (2) more explicitly account for interactions between subpopulations with different dispersal rates.

BOX 1 **Related reaction–diffusion models.**

In Skellam's (1951) seminal reaction–diffusion model, the evolution of a population density function $\Psi \left(x,y\right)$ is described by a partial differential equation that combines simultaneous processes of random‐walk dispersal (with characteristic step size proportional to a constant a) and reproduction (at a constant per‐capita growth rate c>0):

(B1) $$\frac{\partial \Psi }{\partial t}=\frac{a^{2}}{4}\nabla^{2}\Psi +c\Psi ,$$

where $\nabla^{2}=\frac{\partial^{2}}{\partial x^{2}}+\frac{\partial^{2}}{\partial y^{2}}$ is the Laplacian operator. Skellam considered Equation (B1) on a circular domain of radius $r_{0}$, beyond the outer edge of which lie a “zone of absolute extinction,” while enforcing an “absorbing” (i.e., Dirichlet) boundary condition (Pudjaprasetya, 2018) of Ψ=0 along the edge for continuity. Regardless of the population's initial distribution throughout the domain, solutions have a dominant mode consisting of a dome‐shaped density function that grows (or decays) exponentially at a spatially uniform rate $k=c-a^{2}j_{1}^{2}/\left(4r_{0}\right)$ (where $j_{1}\approx 2.405$). Populations with *faster dispersal rates* would traverse the “absorbing” boundary in greater numbers, inhibiting their growth; this lethal boundary effect becomes more exaggerated on smaller domains. For a given domain size $r_{0}$, populations with excessive dispersal rates ($a>2\sqrt{cr_{0}}/j_{1}$) fail to maintain densities sufficient to support net growth, and so decay to extinction; similarly, for a population with a given dispersal rate a, there is a critical patch size ($r_{0}>a^{2}j_{1}^{2}/\left(4c\right)$) below which the population cannot persist.

More general reaction–diffusion models have taken forms such as

(B2) $$\frac{\partial \Psi }{\partial t}=\nabla \cdot a\left(x,y\right)\nabla \Psi +c\left(x,y\right)\Psi ,$$

where the functions $a\left(x,y\right)$ and $c\left(x,y\right)$ can accommodate spatial variations among dispersal rates and per‐capita growth rates, respectively (Cantrell & Cosner, 2004). Boundary conditions were also formulated much more generally, allowing for a hybrid of (partial) absorption and (partial) reflection:

(B3) $$a\left(x,y\right)\frac{\partial \Psi }{\partial \vec{n}}+\beta \left(x,y\right)\Psi =0,$$

where $\vec{n}$ is an outward normal vector with respect to the domain boundary, and the function $\beta \left(x,y\right)$ describes the fraction of individuals impinging upon the boundary at $\left(x,y\right)$ that can traverse it (Cantrell & Cosner, 2004). Treatment of eigenvalue problems that arise from Equations (B2) and (B3) yielded more general conclusions about the overall rates of population growth or decay: the rate of population loss across a boundary is proportional to the largest eigenvalue of the diffusion operator on the domain Ω, which is determined primarily by the patch area and not by irregularities in the shape of the boundary ∂Ω. Even when “no‐flux” (i.e., Neumann) boundary conditions (Pudjaprasetya, 2018) are applied, analyses of source–sink dynamics within the domain lead to predictions regarding critical patch sizes and long‐term persistence (Cantrell & Cosner, 2001, 2004).

The tendency toward *reduced dispersal ability* in spatially‐heterogeneous habitats was studied within a reaction–diffusion framework by Dockery et al. (1998), who considered the coevolution of multiple population density functions $\Psi_{k}$ (for k=1,…,n), each representing a distinct phenotype k with characteristic dispersal rate $a_{k}$, in environments with spatially varying carrying capacities $K\left(x,y\right)$:

(B4) $$\frac{\partial \Psi_{k}}{\partial t}=\frac{a_{k}^{2}}{4}\nabla^{2}\Psi_{k}+\Psi_{k}\left[K\left(x,y\right)-\sum_{i=1}^{n}\Psi_{i}\right]+\epsilon \sum_{i=1}^{n}M_{\mathrm{ki}}\Psi_{i},$$

where ϵ>0 is small and $M_{\mathrm{ki}}$ encodes the relative frequencies of random mutations from phenotype i into phenotype k. Spatial heterogeneity in the environment (i.e., in $K\left(x,y\right)$) was shown to shift the relative abundances of the phenotypes in favor of slower dispersers. In contrast with this study, in which subpopulations interact directly via sexual reproduction, the functions $\Psi_{k}$ in that case were coupled to one another only via the logistic growth term, which depends on total population density. The small linear mutation term was used to confirm the robustness of the model's conclusions with respect to mutations.

A number of studies sought to further develop the pioneering work of Skellam and others (e.g., Kierstead & Slobodkin, 1953) by applying reaction–diffusion models to investigate ecological problems in greater depth (Britton, 1986; Ōkubo et al., 2001); a comprehensive review is given by Cantrell and Cosner (2004). These analyses considered reaction–diffusion dynamics on domains with more general shapes and boundary conditions, while sometimes also accommodating spatial heterogeneity among per‐capita growth rates or dispersal rates within a domain's interior (see Box 1, Equations B2 and B3). These analyses provided a more thorough theoretical understanding of how a habitat's shape, boundaries, and interior source–sink dynamics can affect a dispersing population's long‐term persistence, making predictions about the critical patch sizes required for survival (Cantrell & Cosner, 2001, 2004). These insights were used to formulate more generalized reaction–diffusion approaches toward island biogeography (Cantrell et al., 1996; Cantrell & Cosner, 1994, 2001), while remaining applicable to a wider variety of scenarios of interest in landscape ecology.

Meanwhile, other reaction–diffusion modeling efforts explicitly modeled the interactions between coexisting subpopulations with different dispersal traits. Unlike models that focused on the long‐term persistence of populations of individuals all sharing the same dispersal rate, Dockery et al. (1998) explicitly modeled *variability* among dispersal abilities. Their approach considered the coevolution of multiple population density functions, each representing a phenotype characterized by its own distinct dispersal rate (see Box 1, Equation B4), and coupled to the other phenotypes through competition for resources and small mutations. By tracking how the relative abundances of slower and faster dispersers would change as they dispersed through an environment with spatially varying carrying capacity, the model predicted a universal tendency toward dispersal ability loss (Dockery et al., 1998). Other approaches have since obtained similar results using models formulated with continuous rather than discretized dispersal rates (Lam & Lou, 2017), and ongoing research has continued to use reaction–diffusion approaches that incorporate environmental heterogeneity in new ways (Cantrell et al., 2020; Wickman et al., 2017).

#### 1.2.2 Metapopulation models

The reaction–diffusion models reviewed above often echoed results from *metapopulation models*. These models partition environments into discrete, interconnected sites while modeling the internal dynamics of each site as “well‐mixed.” Metapopulation models were able to incorporate feedbacks between migration rates and the distributions of genes that determine those migration rates (Asmussen, 1983; Balkau & Feldman, 1973; Ludwig & Levin, 1991; Moody, 1981; Nagylaki & Moody, 1980; Olivieri et al., 1995). In models where environmental characteristics were allowed to vary from site to site (Cohen & Levin, 1991; Hastings, 1983; Holt, 1985; Levin et al., 1984; McPeek & Holt, 1992), heterogeneity was repeatedly shown to reduce dispersal abilities (Kirkland et al., 2006; Murrell et al., 2002; Papaïx et al., 2013). In contrast, *temporal* heterogeneity was found to *increase* dispersal ability in some cases (Cohen & Levin, 1991; McPeek & Holt, 1992), foreshadowing similar results from reaction–diffusion models (Hutson et al., 2001).

### 1.3 Allee effects

An *Allee effect* (Courchamp et al., 2008) operates when, at lower population densities, increasing density has a positive effect on fitness and reproduction rates. Aggregation, rather than isolation, becomes advantageous. A *strong* Allee effect applies when the effect can go beyond merely slowing growth rates to cause a net population decline. A variety of mechanisms produce Allee effects; for example, spatial aggregation by animals can facilitate cooperation in hunting, foraging, or defense, while in plants, higher vegetation density can help maintain favorable soil conditions that support further growth (Rietkerk et al., 2004). *Mate‐finding Allee effects* specifically associated with sexual reproduction can arise when, for example, animals in sparsely populated areas seldom encounter potential mates, or as pollen propagated by plants into sparsely populated areas too often fails to reach conspecifics (Davis et al., 2004).

In the context of reaction–diffusion models, per‐capita growth rates (see Box 1) can depend on local population density, with the appropriate mathematical form of density dependence determined by the specific mechanisms at hand (Aronson & Weinberger, 1978; Cantrell et al., 1996; Du et al., 2019; Du & Shi, 2007; Shi & Shivaji, 2006). Allee effects can arise from mate‐finding processes due to the relative rarity of encounters between potential mates in sparsely populated areas (Boukal & Berec, 2002, 2009; Gascoigne et al., 2009; Lutscher et al., 2022; McCarthy, 1997); these often share a mathematical form anticipated by Volterra and later termed a *bimolecular collision model* (Dennis, 1989). In these models. the frequencies of encounters between members of different subpopulations are assumed to be proportional to the product of their densities. Some models separately account for male and female subpopulations by allowing for fluctuating sex ratios (Boukal & Berec, 2002, 2009; Gascoigne et al., 2009). For example, Molnár et al. (2008) assumed the local rates of breeding pair formation to be proportional to the product of local female and male population densities. More recent work has investigated reaction–diffusion dynamics incorporating other types of Allee effects (Du et al., 2019; Wang et al., 2019; Wei et al., 2020), and Allee effects have also been extensively studied using metapopulation approaches (Amarasekare, 1998; Courchamp et al., 1999; Pires & Duarte Queirós, 2019).

This study investigates how mate‐finding Allee effects affect the evolution of highly fragmented populations: *How does the geometry of a population's initial distribution in space determine the dispersal characteristics that evolve?* A novel reaction–diffusion model is applied to a series of numerical test cases, each chosen to highlight how a different geometric aspect of a population's fragmentation in space—the sizes and densities of fragments, the distances between adjacent fragments, or the presence of fragmentation at multiple spatial scales—can shape the dispersal characteristics of populations.

## 2 METHODS

### 2.1 Dynamical equations

We consider the coevolution of n population density functions $\psi_{k}\left(X,Y\right)$, each representing a distinct genotype k distinguished by its own characteristic *dispersal ability* $a_{k}$ (related to the distance traveled per unit time in random‐walk movements). These dispersal abilities take one of n evenly spaced values, $a_{k}=a_{0}k$, for k=1,…,n (where $a_{0}>0$). The environment is assumed to have a finite *carrying capacity* K, such that a logistic growth factor attenuates birth rates as the environment becomes saturated. The mate‐finding process affects birth rates in accord with the assumption that births result from random encounters between *pairs* of individuals (as in bimolecular collision models; Dennis, 1989). By further assuming a constant, spatially uniform sex ratio, the local probability of encounters between members of subpopulations i and j becomes proportional to the product $\psi_{i}\;\psi_{j}$. A factor $\chi_{k}^{\mathrm{ij}}$ describes the probability ($\sum_{k=1}^{n}\chi_{k}^{\mathrm{ij}}=1$) that parents of genotypes i and j will produce offspring of type k. Deaths are assumed to occur randomly with probability d within each time increment. The population density function $\psi_{k}$ representing genotype k thus evolves with respect to time τ according to the dynamical equation

(1) $$\frac{\partial \psi_{k}}{\partial \tau }=\frac{a_{k}^{2}}{4}\nabla^{2}\psi_{k}+b\left[\sum_{i=1}^{n}\sum_{j=1}^{n}\chi_{k}^{\mathrm{ij}}\psi_{i}\psi_{j}\right]\left[1-\frac{1}{K}\sum_{i=1}^{n}\psi_{i}\right]-d\psi_{k},$$

where $\nabla^{2}=\frac{\partial^{2}}{\partial X^{2}}+\frac{\partial^{2}}{\partial Y^{2}}$ is the Laplacian operator and all parameters are positive.

We focus on the case where offspring have an equal 50% chance of inheriting the genotype k of either parent, so that

(2) $$\chi_{k}^{\mathrm{ij}}=\frac{1}{2}\left[\delta \prime_{\mathrm{ik}}+\delta \prime_{\mathrm{jk}}\right],$$

where $\delta \prime_{\mathrm{ij}}=1$ if i=j and $\delta \prime_{\mathrm{ij}}=0$ if i≠j. In this case, Equation 1 becomes

(3) $$\frac{\partial \psi_{k}}{\partial \tau }=\frac{a_{k}^{2}}{4}\nabla^{2}\psi_{k}+\left(\mathrm{b\psi}\left[1-\frac{1}{K}\psi \right]-d\right)\psi_{k}=\frac{a_{k}^{2}}{4}\nabla^{2}\psi_{k}+c\left(\psi \right)\psi_{k},$$

where $\psi =\sum_{i=1}^{n}\psi_{i}$ is the *overall population density*. The net *per‐capita growth rate*,

(4) $$c\left(\psi \right)=\mathrm{b\psi}\left[1-\frac{1}{K}\;\psi \right]-d,$$

is then identical across all genotypes k, depending only upon the *overall* population density ψ. As in some previous models (e.g., Dockery et al., 1998), then, any changes in the relative abundances of the different genotypes can be attributed unambiguously to their different dispersal rates. In contrast to those models, the mate‐finding process modeled here results in a different nonlinear dependence of the per‐capita growth rate upon total population ψ. Specifically, Equation (4) recalls the class of *strong Allee effect* growth terms studied previously for populations with a uniform dispersal rate (e.g., Amarasekare, 1998; Cantrell et al., 1996; Dennis, 1989; Du & Shi, 2007; Wang et al., 2011, 2019). These growth terms exhibit bistable “explosion/extinction” behavior (Du & Shi, 2007; Shi & Shivaji, 2006; Wang et al., 2011), always evolving toward one of the two possible outcomes: (1) successful colonization of the environment (here, $\psi \to K\left(1+\sqrt{1-4d/\left(\mathrm{Kb}\right)}\right)/2$), or (2) extinction (ψ→0). Beyond addressing questions of long‐term population persistence, though, the inclusion of multiple dispersal genotypes here enables us to consider the *distributions* of dispersal ability that evolve from different fragmented initial conditions.

If population densities are expressed as fractions of carrying capacity as $\Psi_{k}=\psi_{k}/K$, spatial variables in terms of a characteristic length scale $r_{0}$ as $x=X/r_{0}$ and $y=Y/r_{0}$, and time as $t={\left(a_{0}/r_{0}\right)}^{2}\tau$, the dynamical equations (Equation 3) are recast in nondimensionalized form as

(5) $$\frac{\partial \Psi_{k}}{\partial t}=\frac{k^{2}}{4}\nabla^{2}\Psi_{k}+\left(\beta \Psi \left[1-\Psi \right]-\delta \right)\Psi_{k},$$

where $\nabla^{2}=\frac{\partial^{2}}{\partial x^{2}}+\frac{\partial^{2}}{\partial y^{2}}$ now, and $\Psi =\sum_{i=1}^{n}\Psi_{i}$ is the rescaled *overall population density*. Two dimensionless parameters remain: a rescaled *birth parameter* $\beta \equiv \left[K{\left(r_{0}/a_{0}\right)}^{2}\right]b$ and rescaled *death rate* $\delta \equiv {\left(r_{0}/a_{0}\right)}^{2}d$. This illustrates that for given a set of initial population configurations, the task of exploring the range of dynamics possible throughout space of all parameter values can effectively be reduced to an exploration over a range of values of the birth parameter β and death rate δ.

### 2.2 Numerical scheme

We simulate the dynamics of Equation (5) using a finite difference method (Pudjaprasetya, 2018), approximating the Laplacian operator $\nabla^{2}$ using a 9‐point stencil (LeVeque, 2007) while applying periodic boundary conditions. We consider a computational grid with node spacing Δx spanning horizontal coordinates $X=\left\{x_{1},\ldots ,x_{N_{x}}\right\}$ and vertical coordinates $Y=\left\{y_{1},\ldots ,y_{N_{y}}\right\}$. Discretized population density states $\Psi_{k}\left(x,y;t=0\right)$ defined on grid points $\left(x,y\right)\in X\times Y$ (Δx=0.02 for Experiments A–D and Δx=0.04 in Experiment E; see below), are advanced in time according to Equation (5) using a Runge–Kutta method of order 5(4). Dispersal ability values are discretized into n=5 bins, a number chosen to balance considerations of computation and visualization with the need to represent a gradation of dispersal rates.

Simulation results are then summarized in terms of the *total population of genotype* k, $P_{k}\left(t\right)=\left[\sum_{i=1}^{N_{x}}\sum_{j=1}^{N_{y}}\Psi_{k}\left(x_{i},y_{j};t\right)\right]{\left(\Delta x\right)}^{2},$ the total *overall population* $P\left(t\right)=\sum_{k=1}^{n}P_{k}\left(t\right),$ and the population's *mean dispersal ability*, $\bar{a}\left(t\right)=\frac{1}{P\left(t\right)}\;\sum_{k=1}^{n}P_{k}\left(t\right)a_{k}.$ Unless otherwise noted, simulations were terminated when the value $\sigma \left(t\right)/\bar{\Psi_{1}}\left(t\right)$ (where σ is the standard deviation of genotype k=1 density values $\Psi_{1}\left(x_{i},y_{j};t\right)$, and $\bar{\Psi_{1}}$ is their mean) receded to below $10^{-3}$ (i.e., when the slowest class of dispersers have nearly achieved a spatially uniform population density), or when the total overall population $P\left(t\right)$ receded to below $10^{-4}$ (extinction). Simulation code is available at https://osf.io/qy5gf/?view_only=9d069efcd76e4379a8a6874b27dd2e4d.

### 2.3 Fragmented population configurations

**FIGURE 1:**
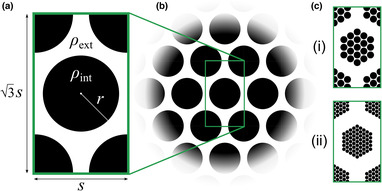
(a) Layout of initial population density functions $\Psi \left(x,y;t=0\right)$ for Experiments A–D. Circular patches of radius r and uniform density $\rho_{\mathrm{int}}$ are positioned as shown within a rectangular domain. Outside these patches, the domain is populated with uniform density $\rho_{\mathrm{ext}}$. (b) Modeling an infinite lattice of population fragments. Due to the use of periodic boundary conditions in simulations, the layout shown in (a) can be interpreted as modeling an infinitely extended tiling of identical fragments. Simulating the dynamics within this domain is thus equivalent to simulating the dynamical evolution of a triangular lattice of circular patches with nearest‐neighbor spacing s. (c) Initial conditions for Experiment E. The computational domain is expanded and intervening exterior areas are introduced atop the regular lattice‐like arrangement of fragments simulated previously such that “micro”‐fragments are grouped into larger “macro”‐fragments, themselves arranged in a triangular lattice configuration. We consider two variations on this layout, both sharing the same overall initial population: (i) *coarse fragmentation* and (ii) *fine fragmentation*.

Rigorous studies of reaction–diffusion models have explored how patch/fragment geometry determines long‐term outcomes by delineating the ranges of patch sizes, densities, or spacings over which survival or extinction will result (Cantrell & Cosner, 2001, 2004). While a similar analytical approach is beyond our scope, this study also systematically explores how different aspects of a population's spatial configuration—fragment sizes, densities, and spacings—affect evolutionary outcomes under mate‐finding Allee effects. To this end, we deal with idealized fragmented populations of which the characteristic sizes, densities, and spacings of fragments can be varied (Figure 1). We detail the layouts of these configurations in the following.

To approximate an equilateral triangular lattice of circular patches with nearest‐neighbor spacing s, grid points $\left(x_{i},y_{j}\right)$ are defined at $x_{i}=-\frac{s}{2}+\Delta x\left(i-1\right)$ from $i=1,\ldots ,N_{x}$ (with $N_{x}=\frac{s}{\Delta x}\;$) and $y_{j}=-\left\lceil \frac{\sqrt{3}}{2}\frac{s}{\Delta x}\right\rceil \Delta x+\Delta x\left(j-1\right)$ for $j=1,\ldots ,N_{y}$ (with $N_{y}=\left\lceil \frac{\sqrt{3}s}{\Delta x}\right\rceil$, where $\left\lceil \cdot \right\rceil$ rounds its argument to the next larger integer). Initial population configurations consist of a circular region of radius r positioned at the center of a rectangular domain, with additional circular quadrants with radii r at each corner (Figure 1a). Dimensions are chosen such that circular region centers are separated by a distance of approximately s. With these initial configurations and periodic boundaries, the layout can be interpreted as representing an infinitely extended triangular lattice of identical circular patches with spacing s (Figure 1b) (while noting that this precludes capturing larger scale spatial phenomena that could develop on true spatially extended domains). Circular region interiors are populated with uniform density $\rho_{\mathrm{int}}$, in which each genotype is represented equally ($\Psi_{k}\left(x,y;t=0\right)=\rho_{\mathrm{int}}/n$ for grid points $\left(x,y\right)$ falling within these regions), while exterior regions are populated with uniform density $\rho_{\mathrm{ext}}$ ($\Psi_{k}\left(x,y;t=0\right)=\rho_{\mathrm{ext}}/n$ for $\left(x,y\right)$ outside these regions). The initial mean dispersal rate is thus $\bar{a}\left(0\right)=\left(1+n\right)/2=3$. A configuration can thus be characterized by four parameters: (1) *Patch radius* r, (2) *lattice spacing* s, (3) *patch interior population density* $\rho_{\mathrm{int}}$, and (4) *patch exterior population density* $\rho_{\mathrm{ext}}$.

For each initial configuration, simulations can be repeated over a range of birth parameter β and death rate δ values to explore how outcomes are affected by environmental conditions. This study comprises five test cases (summarized in Table 1). The first three test cases model sparse, fragmented populations dispersing within an otherwise unpopulated domain ($\rho_{\mathrm{ext}}=0$). The extent of fragmentation is varied from trial to trial (in terms of spacing s in Experiments A and B, and in terms of radius r in Experiment C). The mean dispersal rates $\bar{a}$ that evolve are then observed for those populations that persist. In Experiment D, the layout is inverted to simulate an otherwise‐saturated domain ($\rho_{\mathrm{ext}}=K$) in which circular regions are initially unpopulated ($\rho_{\mathrm{int}}=0$); this can be seen as representing a well‐established population following its disturbance by some spatially localized, catastrophic depopulation events. Experiment E considers populations that are fragmented across multiple spatial scales, with fragments forming a roughly self‐similar lattice of lattices (Figure 1c). Additional details about each of these test cases are discussed alongside simulation results below.

**TABLE 1:** Initial conditions and parameter settings/ranges for the five test cases. Independent variables (i.e., quantities plotted along the horizontal axes in Figure 2) are italicized; Ranges in parentheses (e.g., “(350–400)”) indicate the limits of the parameter regimes over which simulation trials were repeated. Entries for Experiments A through D correspond to the layout of Figure 1a. Entries for Experiment E refer to the (i) *coarse fragmentation* and (ii) *fine fragmentation* layouts displayed in Figure 1c, with S and R indicating the spacing and radius, respectively, of the “macro”‐lattice of hexagonal fragments, with s and r describing the spacings and radii of the circular “micro”‐fragments.

Experiment	Lattice spacing s	Radius r	Interior density $\rho_{\mathrm{int}}$	Exterior density $\rho_{\mathrm{ext}}$	Birth parameter(s) β	Death rate(s) δ
A	*2.0–2.5*	1.0	0.1273	0	365	Interior $\delta_{\mathrm{int}}$: 35 Exterior $\delta_{\mathrm{ext}}$: (10–70)
B	*2.0–2.5*	1.0	0.1273	0	(350–400)	(35–39)
C	2.0	*0.1–0.9*	$\approx \frac{0.1273}{r^{2}}$	0	(350–380)	(45–60)
D	*2.0–2.5*	1.0	0	1.0	(360–380)	(40–55)
E	(i): 2.2 (S=13.2) (ii): 1.1 (S=13.2)	(i): 1.0 (R≈5) (ii): 0.5 (R≈5)	(i): 0.1273 (ii): $\left(\frac{19}{61}\right)0.1273$ ≈.0397	0	385	37

## 3 RESULTS

### 3.1 Experiment A: Dispersal of population fragments between habitat islands

Numerous studies have identified gene flow between population fragments, including cases of “genetic rescue” between habitat islands, as important factors in evolution. Situations like these can be modeled as an “archipelago” of habitat patches separated from one another by regions with inhospitable—but neither strictly lethal nor impenetrable—conditions. Other work has applied reaction–diffusion approaches to investigate related issues of island biogeography or other complex habitats, but has not typically focused on how the initial arrangements of population fragments—as distinct from habitat fragments—might affect the extent of the subsequent changes in dispersal ability. Experiment A uses this “habitat islands” scenario to clarify and distinguish the potential roles of habitat and population fragmentation in complex scenarios like these. Its results provide context for the spatially homogeneous test cases that follow.

**FIGURE 2:**
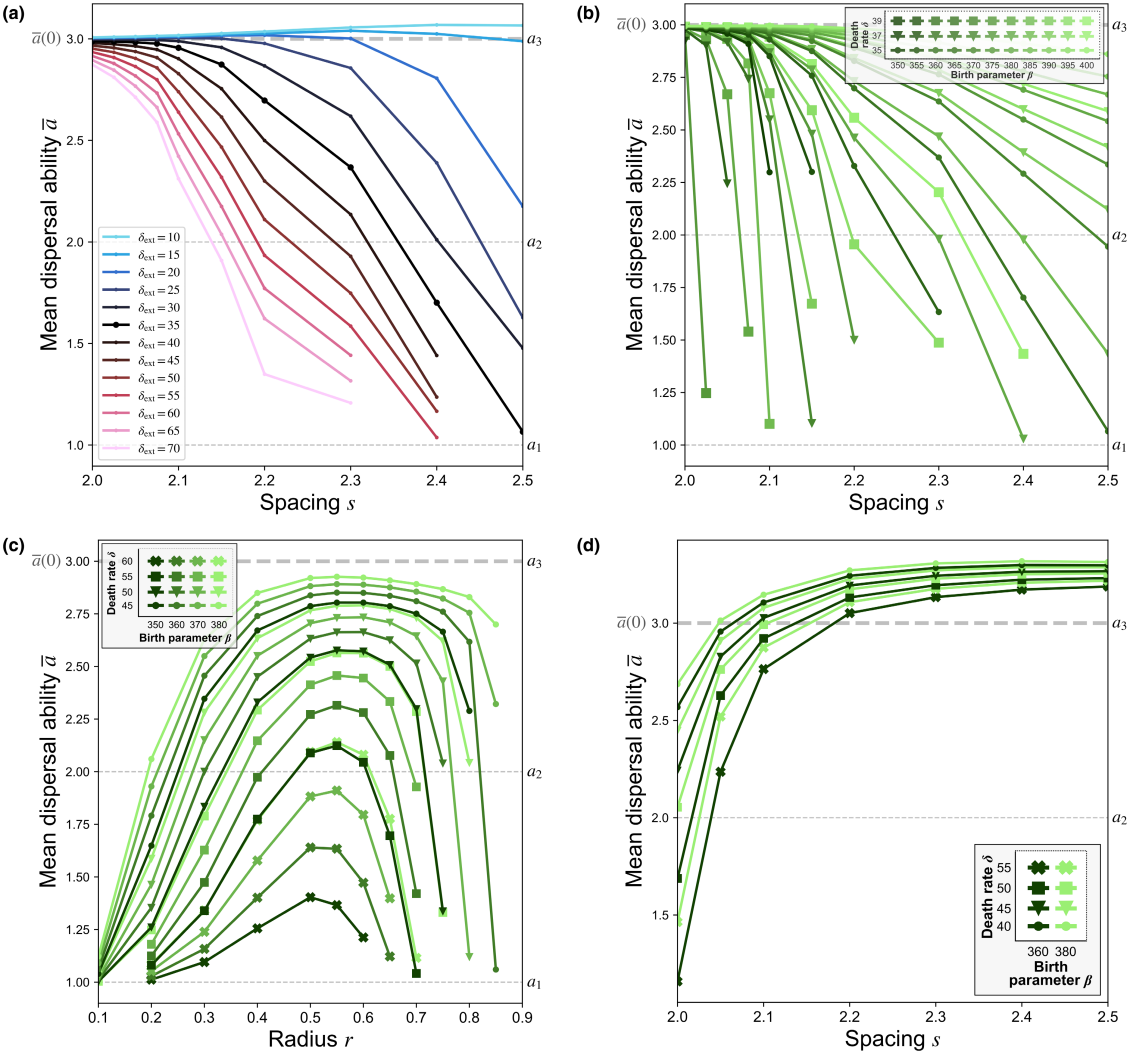
Final population mean dispersal rate $\bar{a}$ as a function of the independent variable characterizing the initial conditions in each test case. (a) Experiment A. Final mean dispersal ability $\bar{a}$ as a function of spacing s for various values of the patch exterior death rate $\delta_{\mathrm{ext}}$ (with $\delta_{\mathrm{int}}=35$ and β=365). (b) Experiment B. Final mean dispersal ability $\bar{a}$ as a function of lattice spacing s for surviving populations over a range of combinations of birth parameter β and death rate δ. (c) Experiment C. Final mean dispersal ability $\bar{a}$ as a function of patch radius r for surviving populations. (d) Experiment D. Final mean dispersal ability $\bar{a}$ as a function of lattice spacing s. Data points representing simulations which resulted in extinction are omitted.

Circular population fragments, initially populated with density $\rho_{\mathrm{int}}$ (Figure 1a), are set to coincide with circular *habitat islands* wherein $\delta \left(x,y\right)=\delta_{\mathrm{int}}$, with initially unpopulated exterior regions where $\delta \left(x,y\right)=\delta_{\mathrm{ext}}$. While holding patch radii r, initial interior and exterior densities $\rho_{\mathrm{int}}$ and $\rho_{\mathrm{ext}}$, and parameters β and $\delta_{\mathrm{int}}$ constant across all trials (see Table 1), we repeat simulations over a range of values of patch‐exterior death rate $\delta_{\mathrm{ext}}$. The final mean dispersal rates $\bar{a}$ shown in Figure 2a are the values achieved when the condition $\left|\sum_{i=1}^{N_{x}}\sum_{j=1}^{N_{y}}\frac{{\partial \Psi }_{k}}{\partial t}\left(x_{i},y_{j};t\right)\right|{\left(\Delta x\right)}^{2}<0.1$ is first satisfied for all k; these do *not* represent steady states, but rather the states attained soon after the habitat has become saturated and Allee effects have ceased to play a primary role.

The curve representing the homogeneous environment case ($\delta_{\mathrm{ext}}=\delta_{\mathrm{int}}=35$) in Figure 2a shows how the final mean dispersal rate $\bar{a}$ decreases steadily as initial spacings s are increased. For less‐hospitable values of the patch exterior death rate ($\delta_{\mathrm{ext}}>\delta_{\mathrm{int}}$), the circular regions represent *habitat islands* with more favorable conditions embedded within a less hospitable exterior. The presence of higher mortality in the exterior enhances the dispersal ability loss that occurs due to Allee effect losses alone in a homogeneous environment; curves show a qualitatively similar, but more exaggerated, dependence of final dispersal ability upon lattice spacing s. When population fragments initially anchored to habitat islands propagate outward, the transient dynamics of interest are largely captured by the homogeneous environment case; spatial heterogeneity in death rates boost or hinder these dynamics. If these population fragments instead propagate outward into regions where mortality is lower ($\delta_{\mathrm{ext}}<\delta_{\mathrm{int}}$), then competition to occupy the exterior region can become more important than mate‐finding Allee effects in driving selection, so that faster dispersal is advantageous. These results reveal the potential for mean dispersal ability to evolve *upward* in this model (as when $\delta_{\mathrm{ext}}=10$ in Figure 2a), marking an important qualitative difference between the dynamics that result from population fragmentation from those associated with habitat fragmentation.

Over the longer run, however, spatial gradients in the death rate $\delta \left(x,y\right)$ will continue to drive a net flux of dispersers into the more lethal regions. Genotypes that disperse more quickly into these less hospitable regions will be disproportionately affected, gradually draining the population of its more rapid dispersers. The mean dispersal abilities that initially result from Allee effects (Figure 2a) will not persist in the long run. When habitat and population fragmentation coincide, the heterogeneity‐driven dispersal ability loss observed in so many previous models will indeed occur here. However, since these habitat‐driven changes can be orders of magnitude slower than those that result from Allee effects, the dispersal traits that initially evolve due to fragmentation can endure for a relatively long time. For example, in the simulation with the most heterogeneous environment considered here ($\delta_{\mathrm{ext}}=35$, $\delta_{\mathrm{int}}=10$, s=2.2), mean dispersal ability attains the value displayed in Figure 2a as the environment becomes saturated around t≈0.1, while by t≈6, the effects of the heterogeneous environment have only reduced mean dispersal ability to 90% of this previous value. Allee effect‐driven processes could thus remain ecologically relevant for a long time, especially in less heterogeneous environments, or in cases where additional fragmentation can occur over time. These observations provide motivation and context for our focus on *population fragmentation*, rather than *habitat fragmentation*, in the homogeneous environment test cases that follow.

Animations of the simulations of Experiment A summarized in Figure 2a are accessible at https://osf.io/49863/?view_only=e81267c03e474f16b551237c27fa3d74.

### 3.2 Experiment B: The role of separation between population fragments

**FIGURE 3:**
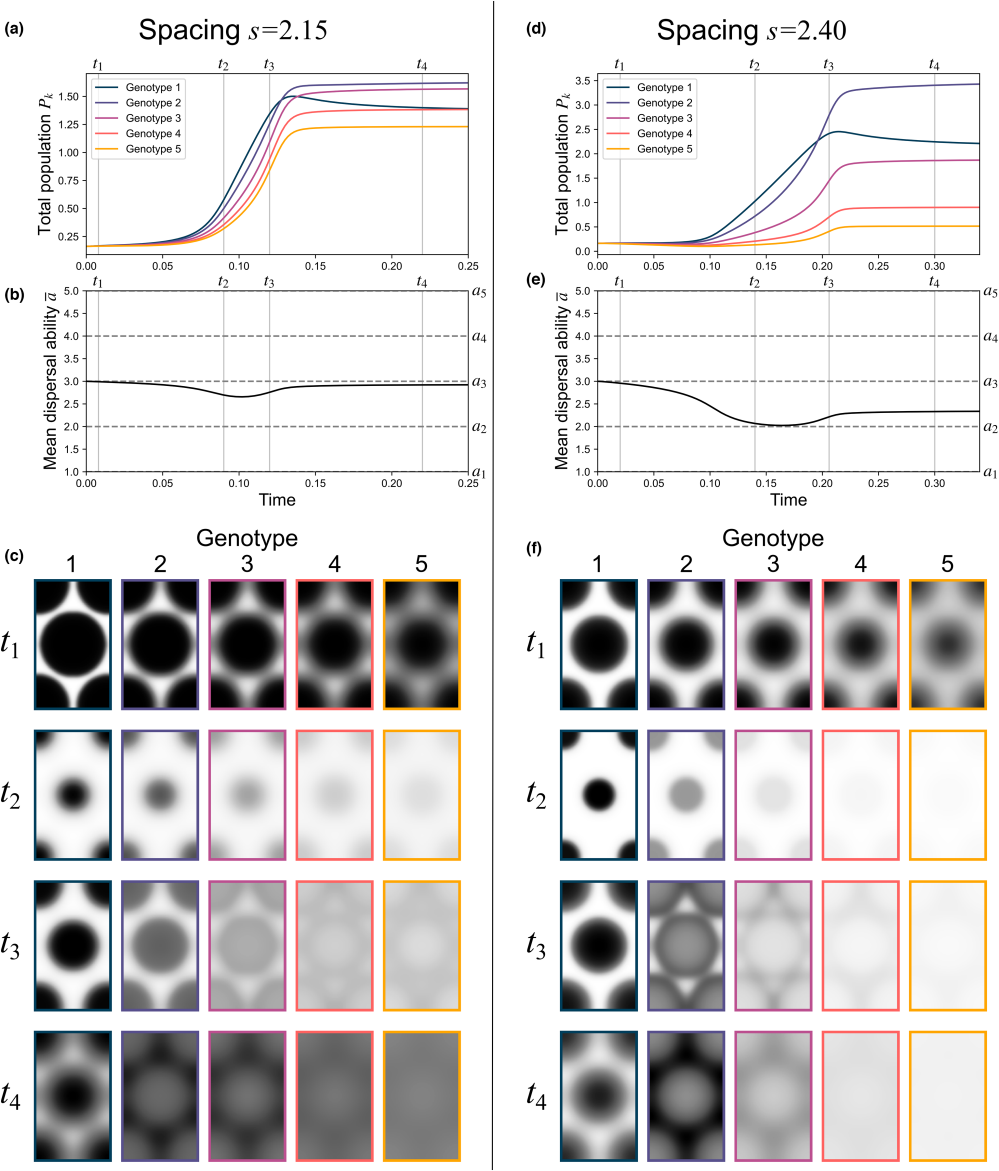
Simulation results from Experiment B for lattice spacings s=2.15 (left column) and s=2.40 (right column) with β=380, δ=36. **(**a) Time evolution of the total population of each genotype for spacing s=2.15. (b) Time evolution of population mean dispersal ability $\bar{a}$ for spacing s=2.15. (c) Relative population densities $\Psi_{k}\left(x,y;t\right)/\Psi_{\max}\left(t\right)$ (where $\Psi_{\max}\left(t\right)=\max_{x,y,k}\;\Psi_{k}\left(x,y;t\right)$) at indicated times $t_{1}$, $t_{2}$, $t_{3}$, and $t_{4}$ for spacing s=2.15. (d) Time evolution of the total population of each genotype for spacing s=2.40. (e) Time evolution of population mean dispersal ability $\bar{a}$ for spacing s=2.40. (f) Relative population densities $\Psi_{k}\left(x,y;t\right)/\Psi_{\max}\left(t\right)$ at indicated times $t_{1}$, $t_{2}$, $t_{3}$, and $t_{4}$ for spacing s=2.40. Note that by the times $t_{4}$ shown here, the relative abundances of each genotype have nearly reached their steady‐state values; following $t_{4}$, populations continue to diffuse, approaching spatially uniform distributions.

Experiment B retains the initial population configurations used in Experiment A, but now considers their dispersal within *spatially homogeneous* environments over a range of combinations of birth and death parameters. While holding the initial sizes, shapes, and densities of these patches consistent across all trials, we run a series of simulations that vary the spacing s separating the fragment centers. Lower values of s give more closely packed fragments; as s is increased, these fragments become increasingly isolated from one another. As simulations proceed from these fragmented initial conditions ($t_{1}$ in Figure 3), faster dispersers propagate first into these unpopulated regions, and so are more severely affected by Allee effects; meanwhile, slower dispersers remain densely aggregated around fragment centers, where they maintain higher growth rates ($t_{2}$ in Figure 3). When fragments are more closely spaced, dispersers from adjacent fragments more quickly meet and establish higher population densities in the gaps between patches ($t_{3}$ in Figure 3). This reduces the losses of rapid dispersers suffered early on, retaining more of their genes within a population that (given sufficiently hospitable conditions) then proceeds to saturate the environment ($t_{4}$ in Figure 3). However, when fragments are separated by larger spacings s, the greater times taken to traverse the intervening gaps results in greater losses of rapid dispersers before overall population numbers stabilize. Once the population has established itself in greater numbers, surviving rapid dispersers may regain some advantage as they are able to occupy the sparsely populated regions between fragments first ($t_{3}–t_{4}$ in Figure 3c,f). By initially dominating these regions, rapid dispersers can partially recover the dispersal ability lost during more precarious stages of evolution ($t_{2}–t_{3}$ in Figure 3).

Even as a population saturates the environment, and its constituent genotypes become uniformly intermixed in space, the trauma of the initial fragmentation nonetheless remains “frozen in” to the population, having determined the relative abundances of different genotypes. The mean dispersal ability values attained decrease as the initial spacing s increases (Figure 2b). If the initial spacing s is further increased, then Allee effects become insurmountable and the entire population decays. Under more favorable environmental conditions (i.e., higher ratios β/δ), Allee effect dispersal ability losses (and, eventually, extinctions) begin to take effect at larger spacings s; the more favorable the conditions, the greater the amount of initial spatial isolation that can be overcome.

Animations for the simulations summarized in Figure 3 are shown in Movies S1 and S2, and animations for the simulations of Experiment B, representing the range of parameter values of s, β, and δ included in Figure 2b, are accessible at https://osf.io/2qn8u/?view_only=7aa42a040f0b417aa571266a0e465a9d.

### 3.3 Experiment C: The role of fragment size and density

In Experiment B, the spacing s between fragment centers was used to track the effects of spatial separation upon the dispersal abilities that evolved. Increasing the patch spacing s in this way also results in an increased domain *area* (Figure 1a), which decreases the population's initial overall density (i.e., the ratio of total overall population to domain area ($\approx P/\left(\sqrt{3}s^{2}\right)$)). Experiment C aims to disentangle the geometric aspects of fragmentation from variations to the overall population density by varying patch size r in tandem with density $\rho_{\mathrm{int}}$, so that the geometry of the initial configuration is altered without affecting its initial overall density.

Many results show significant reductions in mean dispersal ability $\bar{a}$, with dynamics qualitatively similar to those observed in Experiment B (Figure 2c). The observed decline in mean dispersal rates as initial patch radii r are decreased (i.e., progressing leftward in Figure 2c) corresponds to the decline observed for increasing spacing s in Experiment B: in both cases (as s is increased, or r is decreased), the unpopulated gaps between fragments are expanded. Here, however, the non‐monotonic dependence of the final mean dispersal rate $\bar{a}$ upon r (Figure 2c) illustrates the trade‐off between high population density *within* fragments (which enhances a fragmented population's ability overcome Allee effects during the precarious initial stages of evolution) and smaller intervening distances *between* fragments (which facilitates the rapid colonization of unpopulated areas, reducing Allee effects there). Small, densely aggregated fragments are more spatially isolated; despite their high interior densities, these populations suffer great reductions in dispersal ability (or extinction) as they struggle to populate the vast surrounding regions. On the other hand, when fragments are wider but less densely aggregated, populations may be spread too thin to overcome Allee effects even within fragments. Between these extremes, the advantages of closer spacings between fragments (which support the survival of faster dispersers) and higher densities within fragments (where slower dispersers stay put and reproduce) complement one another. All else being equal, an *intermediate* degree of spatial fragmentation here enhances the chances of survival compared to more‐ or less‐fragmented initial configurations, including for more rapid dispersers who disproportionately suffer Allee effects at both extremes.

Animations of the simulations of Experiment C summarized in Figure 2c are accessible at https://osf.io/mtbc4/?view_only=05ef0d96d6224671b098cb107bc872a1.

### 3.4 Experiment D: Disruption of established populations by localized depopulation

Even dense populations can become vulnerable to Allee effects if they are affected by some spatially localized disturbance (e.g., forest fires or human‐induced deforestation; Brisson et al., 2003). Experiment D inverts the configurations of the previous test cases by situating unpopulated circular patches among a densely populated exterior region ($\rho_{\mathrm{ext}}=K$, $\rho_{\mathrm{int}}=0$), as if patch interiors had just been suddenly depopulated. Changes to the spacing s alter the spatial extent of these disruptions. For smaller spacings s, a majority of the domain has been depopulated, and fragments disperse outward from narrow, densely populated slivers. These isolated population fragments struggle to repopulate the vast unpopulated regions, and so experience Allee effect dispersal ability losses (Figure 2d) like those observed in previous cases. For larger spacings s, only distant pockets have been depopulated; the population—including all of its constituent genotypes—remain safe from Allee effects. Under these more favorable conditions, Allee effect losses are less severe, and the clearing‐out of previously saturated regions favors rapidly dispersing pioneers who manage to establish their presence there first, leading to net *gains* in mean dispersal ability for larger spacings s (Figure 2d). Along the propagating front of expanding population fragments, rapidly dispersing pioneers make their way outward into the unpopulated void; not far behind them, though, slower dispersers maintain higher densities, reproducing with faster dispersers to ensure a constant feed of new rapid dispersers to act as pioneers. This result echoes empirical and theoretical studies that investigate how *increased* dispersal ability can evolve along the range edges of expanding population fronts (Bénichou et al., 2012; Bouin et al., 2012; Deforet et al., 2019; Hughes et al., 2007; Léotard et al., 2009; Phillips, 2009; Phillips et al., 2010; Simmons & Thomas, 2004; Tobin et al., 2007; Travis et al., 2009, 2010; Travis & Dytham, 2002).

Animations of the simulations of Experiment D summarized in Figure 2d are accessible at https://osf.io/xg6z7/?view_only=be10651f66e24f2ba4fb85ed4ff4118c.

### 3.5 Experiment E: Fragmentation across multiple spatial scales

The idealized fragmented configurations of the previous test cases demonstrate how the spatial separations, densities, and sizes of identical fragments affect the extent of dispersal ability losses (or gains) that evolve due to mate‐finding Allee effects. More realistic populations can contain fragmentation at multiple spatial scales. Experiment E considers more elaborate configurations where fragments are grouped into larger macro‐fragments, which themselves are also arranged in a triangular lattice (Figure 1c). We consider two different population configurations that differ only in terms of their spatial fragmentation at the microscale. Both feature large hexagonal regions with “radii” of around 5 and lattice spacings of 13.2, and the same total initial population. In the first configuration, these macro‐fragments are more coarsely subdivided (with r=1.0 and s=2.2); in the second, they are more finely subdivided (with r=0.5 and s=1.1).

**FIGURE 4:**
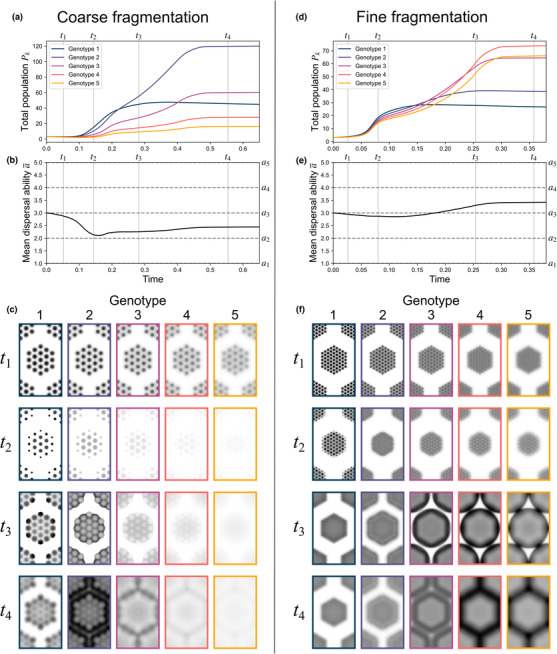
Simulation results from Experiment E with β=385, δ=37. (a) Time evolution of the total population of each genotype for the *coarse fragmentation* case. (b) Time evolution of population mean dispersal ability $\bar{a}$ for the *coarse fragmentation* case. (c) Relative population densities $\Psi_{k}\left(x,y;t\right)/\Psi_{\max}\left(t\right)$ (where $\Psi_{\max}\left(t\right)=\max_{x,y,k}\;\Psi_{k}\left(x,y;t\right)$) at indicated times $t_{1}$, $t_{2}$, $t_{3}$, and $t_{4}$ for the *coarse fragmentation* case. (d) Time evolution of the total population of each genotype for the *fine fragmentation* case. (e) Time evolution of population mean dispersal ability $\bar{a}$ for the *fine fragmentation* case. (f) Relative population densities $\Psi_{k}\left(x,y;t\right)/\Psi_{\max}\left(t\right)$ at indicated times $t_{1}$, $t_{2}$, $t_{3}$, and $t_{4}$ for the *fine fragmentation* case. Note that by the times $t_{4}$ shown here, the relative abundances of each genotype have nearly reached their steady‐state values; following $t_{4}$, populations continue to diffuse, approaching spatially uniform distributions.

Simulating these two configurations under the same combination of parameter settings offers a glimpse into how meta‐scale fragmentation can lead to qualitatively different patterns of evolution than were observed on the simpler layouts considered previously. This is visible in the unique patterns of spatial propagation of the macro‐fragment populations, which differs from those observed in the uniform circular fragments of the previous experiments. Internal fragmentation within the macro‐fragments leads to dispersal ability loss within their interiors, and even greater losses are experienced near the macro‐fragments' outer edges as rapidly dispersing pioneers propagate into unpopulated regions. This results in a relative lack of rapid dispersers around the edge ($t_{2}$ in Figure 4c,f); as the population then builds in overall numbers, however, a surge of new population then propagates outward, driven by rapid dispersers, breeding with the slow dispersers near the edge to produce a greater abundance of slower dispersers around the macro‐fragment edge than is found in the center ($t_{3}$ in Figure 4c,f).

Despite both configurations sharing a similar pattern of fragmentation at the macro‐scale, these two cases differ in terms of their eventual qualitative outcomes. As macro‐fragment populations colonize the intervening unpopulated regions, the more coarsely fragmented population experiences a net dispersal ability *decrease* (Figure 4a), while the more finely fragmented population (r=0.5, s=1.1) experiences a net dispersal ability *increase* (Figure 4b). In the coarsely fragmented case, the population experiences greater initial dispersal ability loss, with mean dispersal ability reaching its lowest level around $t_{2}$ (Figure 4a). Although these relative losses are later partially recovered ($t_{3}$ in Figure 4a) by rapid dispersers colonizing the larger intervening regions between both macro‐ *and* micro‐fragments ($t_{3}$ in Figure 4c), dynamics eventually stagnate with a net dispersal ability decrease having occurred ($t_{4}$ in Figure 4b,c). In the more finely fragmented case, early dispersal ability losses are less extreme due to the narrower gaps between micro‐fragments, which mitigate Allee effect losses ($t_{2}$ in Figure 4e,f). Greater numbers of rapid dispersers are thus retained within the population, and these rapid dispersers have an advantage as well‐established macro‐fragments propagate outward to colonize the surrounding regions, where rapid dispersers come to dominate slower dispersers ($t_{3}$ and $t_{4}$ in Figure 4a–c), leading to a net dispersal ability increase (Figure 4d–f). The effects of the population's initial micro‐fragmentation are thus still felt even long after its traces are no longer visible in the population's spatial distribution ($t_{4}$ in Figure 4d). These effects are manifest not just in the magnitude of dispersal change, but potentially also in its direction: upward or downward.

Full animations for the simulations summarized in Figure 4 are shown in Movies S3 and S4.

## 4 DISCUSSION

The model presented here combines several features of previous reaction–diffusion models in a novel way to study the evolution of dispersal by fragmented populations with an Allee effect. Its use of multiple interacting dispersal ability genotypes distinguishes it from other related reaction–diffusion models that included Allee effects (Cantrell et al., 1996; Shi & Shivaji, 2006). As in the work of Dockery et al. (1998), variability among dispersal abilities is modeled by discretizing the space of possible dispersal rates into several bins, each represented by its own characteristic dispersal ability ($a_{k}$), and then considering the coevolution of the population density functions ($\Psi_{k}$) that represent these subpopulations. In that model, direct interactions between different kinds of dispersers were not a prominent driver of evolution in their own right. Here, however, nonlinear interactions between these different genotypes play a more formative role in the evolution of dispersal traits.

Our results demonstrated how larger distances between population fragments lead to greater dispersal ability losses for small populations that survive to colonize a habitat (Experiment B). For populations with low, fixed initial densities, we demonstrated that an intermediate level of aggregation into fragments is optimal; at lower *or* higher degrees of fragmentation, dispersal ability losses become increasingly severe (Experiment C). When densely populated fragments colonize unpopulated regions, dispersal abilities can evolve upward under favorable conditions (Experiment D). For different model populations showing similar levels of fragmentation at one spatial scale, different patterns of fragmentation at another spatial scale can lead them toward divergent outcomes (Experiment E).

Reaction–diffusion models have long been used to demonstrate how—for populations confronted with heterogeneous habitats or hostile boundaries—slow dispersal can come to dominate over time. The simulations presented here illustrate a case where multiple coexisting strategies—slower dispersers who remain anchored around dense population fragments, and rapid dispersers who colonize sparsely populated areas—can complement one another. Each contributes potential advantages at different phases of evolution; the relative magnitudes of these advantages depend on the distribution of the population in space. This sensitivity to initial conditions is another qualitative difference distinguishing the present model from a great deal of previous related work. Following Skellam (1951), many analytical reaction–diffusion approaches to dispersal have tended to focus only on the long‐term outcomes approached after all traces of initial conditions have vanished. Our results, however, provide examples of highly path‐dependent dispersal evolution, where transient dynamics determine the states that a population approaches in the long run. Even in homogeneous environments, where previously studied mechanisms eventually act to reduce dispersal ability, the consequences of these earlier stages of evolution can remain relevant over relatively long‐time scales (Experiment A).

Within the current model, the details of this fragmentation can even determine the direction—downward or upward—in which dispersal ability evolves. This capacity for predicting upward evolution of dispersal rates under some conditions, even within an unchanging habitat, marks an interesting qualitative distinction of this model from the majority of related work. A few simple mechanisms here can lead either to downward or upward changes in dispersal ability depending on fragmentation patterns and environmental conditions. Given that the purportedly universal *loss of dispersal ability* on islands has been called into question even for the most passive of dispersers (Burns, 2018; García‐Verdugo et al., 2017), models like this could be useful when navigating these issues. Some of the simulation results presented above exhibit features which recall various empirical results reported elsewhere, such as the *upward* evolution of dispersal ability along the expanding fronts of population fragments, which in some cases have been attributed to Allee effects (e.g., Tobin et al., 2007). Further work could explore the possibility of establishing more direct links between models and simulations like those of this study and more concrete empirical observations.

These numerical experiments have explored the space of potential dynamics that can result from population fragmentation and Allee effects, demonstrating some qualitative differences of these dynamics from those of habitat fragmentation. A rigorous theoretical treatment of this model, more akin to that undertaken for other reaction–diffusion models (Cantrell & Cosner, 2004), could provide deeper insights. One advantage of this study's computational approach, though, is that simulation results allow for visualizations of the complex patterns of spatial propagation undergone by multiple interacting classes of dispersers alongside the population‐level genetic changes in dispersal ability that result (as in Figures 3 and 4 and Movies [Link], [Link]). The real utility of such a model may lie in its application to more realistic and specific case studies, where the spatial patterns it predicts could be compared with observed spatial distributions. Previous work has applied related models to problems of conservation or pest control (e.g., Boukal & Berec, 2009; Du et al., 2019); in applications like these, simulations could be useful to help guide spatially targeted interventions. The modeling and simulation approach presented here, by providing visualizations of complex coupled processes of spatial propagation and genetic evolution, could help to facilitate these kinds of connections between models and applications in ways that more abstract, theoretical results alone might not.

## AUTHOR CONTRIBUTIONS

**Poong Oh:** Formal analysis (supporting); supervision (supporting); visualization (supporting); writing – review and editing (supporting). **Matthew F. Jones:** Conceptualization (equal); formal analysis (equal); investigation (equal); methodology (supporting); software (supporting); visualization (equal); writing – original draft (equal); writing – review and editing (equal). **Andrew J. Schauf:** Conceptualization (equal); formal analysis (equal); investigation (equal); methodology (lead); software (lead); visualization (equal); writing – original draft (equal); writing – review and editing (equal).

## FUNDING INFORMATION

Funding was provided by Nanyang Technological University [Grant No. 04INS000411C440], the University of Kansas, and the David B. Jones Foundation.

## Supporting information


Movie S1

Movie S2

Movie S3

Movie S4

## Data Availability

The data that support the findings of this study are openly available from the Open Science Framework at https://osf.io/qy5gf/?view_only=9d069efcd76e4379a8a6874b27dd2e4d.
