# Supplementary figures and images for: Simulating the dynamics of dispersal and dispersal ability in fragmented populations with mate‐finding Allee effects
Source: Ecol Evol. 2023 Apr 21;13(4):e10021. doi: 10.1002/ece3.10021 (PMC10121235; doi:10.1002/ece3.10021)

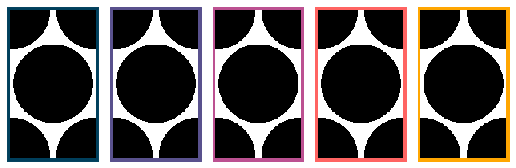

Supplement: Supplementary file 1 — Movie S1 [file ECE3-13-e10021-s002.gif]

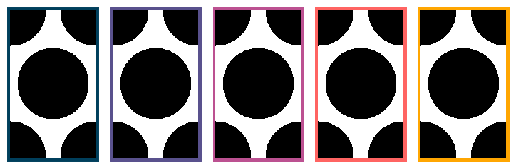

Supplement: Supplementary file 2 — Movie S2 [file ECE3-13-e10021-s004.gif]

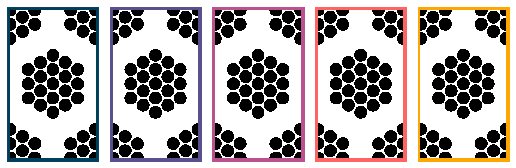

Supplement: Supplementary file 3 — Movie S3 [file ECE3-13-e10021-s003.gif]

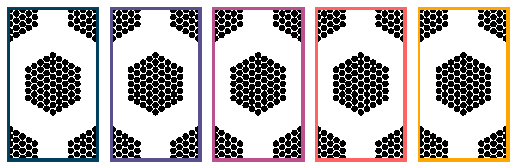

Supplement: Supplementary file 4 — Movie S4 [file ECE3-13-e10021-s001.gif]
